# Flow Cytometry Study of Immune Cell Subpopulations from the Mouse Vertebral Bone Marrow and Intervertebral Disc Following Endplate Microfracture

**DOI:** 10.32604/biocell.2026.074572

**Published:** 2026-04-29

**Authors:** Dalin Wang, Mingcai Zhang, Richard Hastings, Patrick George, Ryan Ranzau, Jinxi Wang

**Affiliations:** 1 The Harrington Laboratory for Molecular Orthopedics, Department of Orthopedic Surgery, University of Kansas Medical Center, 3901 Rainbow Boulevard, Kansas City, KS, USA; 2 Flow Cytometry Core Laboratory, University of Kansas Medical Center, 3901 Rainbow Boulevard, Kansas City, KS, USA; 3 Department of Biochemistry and Molecular Biology, University of Kansas Medical Center, 3901 Rainbow Boulevard, Kansas City, KS, USA

**Keywords:** Flow cytometry, immune cell subsets, bone marrow, intervertebral disc, cartilaginous endplate, spine

## Abstract

**Objective:**

Although endplate (EP) injury may cause intervertebral disc (IVD) degeneration and Modic changes (MCs) in the vertebral bone marrow (VBM), EP injury-induced synchronous cellular reactions and their crosstalk in the IVD and VBM remain unclear. This protocol-based study aimed to streamline and optimize the methods of tissue harvest and cell preparation for flow cytometry (FCM) analysis of T-cell and macrophage subpopulations in both VBM and IVD adjacent to the surgically induced EP microfracture in mice.

**Methods:**

EP injury or sham procedure was performed at the spinal levels L4-5 and L5-6 in male mice. Step-by-step techniques on the harvest of lumbar VBM and IVD tissues, isolation of tissue-specific cells, and generation of single cell suspensions were described. FCM analysis was performed using specific antibodies against cell-surface markers and multi-color cytometry for precise delineation of cell subsets to explore the cellular mechanism of MCs. An extracellular staining assay to identify macrophage subsets, as well as extra- and intra-cellular staining assays to identify T lymphocyte subsets from the VBM and IVD, were performed and optimized.

**Results:**

FCM analysis demonstrated that significantly increased macrophage numbers and M2a polarization response were observed in VBM samples from the sham and EP injury groups, while the ratio of M2a/total number of macrophages was significantly increased and the ratio of M1/M2a was significantly decreased in IVDs from the sham and EP injury groups. A significantly increased Treg cell response was detected in VBM samples from the EP injury group, but not the sham group.

**Conclusions:**

This protocol reports novel and reproducible methods of tissue harvest, cell preparation, and antibody selection for flow cytometry analysis of T-cell and macrophage subpopulations isolated from the VBM and IVD following EP injury in mice. This protocol may be utilized for exploring the cellular mechanism of MCs and IVD degeneration in animal models.

## 1 Introduction

Lowbackpain (LBP) isamajor cause of chronicdisability andhealth care cost burden in theworld [1-3]. LBP can be a result of intervertebral disc (IVD) degeneration, facet joint inflammation and osteoarthritis, and spinal cartilaginous endplate (CEP/EP) injury [4-7]. Modic changes (MCs), characterized by inflammatory changes in the subchondral vertebral bone marrow (VBM) and other musculoskeletal disorders around the spine, may also cause LBP [8-10]. The etiopathogenesis of MCs remains poorly understood [11-13]. Structural damage (e.g., vertebral body burst fracture, accumulating microfracture) to the EP has been proposed as a major cause for MCs, but it cannot fully explain why some patients with EP injury develop MCs while some do not [14-17]. Other disorders, such as autoimmune response of the VBM against the nucleus pulposus (NP), bacterial infections, and genetic changes, are plausible etiologies for MCs [12,17,18].

Previous studies revealed increased infiltration of macrophages in a rat model of MCs [19,20], increased T cells and upregulated expression of interleukin-4 (IL-4) and IL-17 in other MCs models [21,22], and enhanced T helper 1 (Th1)/Th2 and Th17 cell differentiation in LBP patients with type-1 MCs [23]. The polarization of macrophage and CD4^+^Th cells may be involved in the progression of IVD degeneration [24-27]. All models have demonstrated that CD4^+^Th lymphocytes might play a pivotal role in the pathology of MCs and IVD degeneration. Since the vast majority of these studies utilized tissue specimens harvested from the injured or degenerative IVDs, the cellular and tissue interactions between the IVDs and adjacent VBM remain unknown.

It has been proposed that EP structural damage might be an essential prerequisite for triggering an autoimmune response due to interactions of IVD with EP and nearby subchondral bone marrow. This might result in painful MCs and IVD degeneration through a crosstalk between the IVD and VBM [19,23]. However, it is difficult to test and confirm this hypothesis in human patients due to technical challenges in harvesting both IVD and adjacent VBM specimens from the same patient at different time points, which warrants the use of *in vivo* animal models.

Immunohistochemistry (IHC) and reverse transcription polymerase chain reaction (RT-PCR) were used to detect the expression of immune-inflammatory cell lineages in the IVD or adjacent VBM tissue. However, these methods lack the capacity to identify multiple different cell types and their phenotypes simultaneously. With the development of a myriad of surface markers for various immune-inflammatory cell lineages, phenotypes of immune-inflammatory cells can now be reliably identified by distinct combinations of these markers by flow cytometry (FCM) analysis. This technology allows simultaneous identification and quantification of a multitude of immune-inflammatory cells at the single-cell level.

The objective of this protocol-based study was to streamline and optimize the methods of tissue harvest and cell preparation for flow cytometry analysis of T-cell and macrophage subpopulations in both VBM and IVD of the spinal segments with EP microfracture.

## 2 Materials and Methods

### 2.1 Animal Model

This experimental protocol was approved by the Institutional Animal Care and Use Committee at the University of Kansas Medical Center, Kansas City, Kansas, USA (Protocol Number: 22-12-283; date of approval: 02 May 2024). BALB/c strain mice were purchased from the Jackson Laboratory (Bar Harbor, ME, USA) and subsequently housed and cared for in accordance with the Guide for the Care and Use of Laboratory Animals. Male wildtype mice (BALB/c strain), 2.5–3.0 months old, were utilized for this methodological study because the vertebral body and IVD are larger in males than in females, making male mice more suitable for the EP surgical procedure. A total of 30 mice were used in this study for feasibility tests, method optimization, and final FCM analyses.

Animals were divided into EP injury, sham injury, and non-injury naïve groups. Four mice per group were used for feasibility tests and method optimization; six mice per group were used for the final cell preparation and FCM analyses. To induce Modic Changes in mice, the EP microfracture procedure was performed through a similar procedure used for EP microfracture in rats by Wang et al. [19]. Briefly, surgical procedures were performed under aseptic conditions and general anesthesia via inhalation of isoflurane. An anterior abdominal incision was used to expose the L4~L6 lumbar spine. Animals underwent either a sham injury procedure or an EP injury procedure on the L4-5 and L5-6 IVDs. For the sham injury, vertebral bodies (VBs) between L4 and L6 as well as IVD levels L4-5 and L5-6 are exposed, and the cephalad VBs of L4-5 and L5-6 are punctured obliquely at 0.8 mm cephalad to the edge of IVDs using a 25 G needle controlled with a 0.5 mm depth stopper to allow for a shallow vertebral body injury that did not penetrate the EP. For the EP injury, the cephalad EPs of L4-5 and L5-6 were punctured obliquely from the vertebral body at 0.8 mm cephalad to the edge of the IVDs using a 25 G needle controlled with a 1.5 mm depth stopper to allow for the puncture of the EP. The anatomic location of EP injuries was guided and confirmed intraoperatively using the Merry X-ray Imaging system (The Imaging Solutions Company, San Diego, CA, USA).

### 2.2 Isolation of Murine Lumbar Bone Marrow and Intervertebral Disc Cells

To study the cellular and molecular mechanisms of MCs, it is crucial to harvest both the injured IVD and adjacent VBM tissues as follows:

1. Euthanize the animal at 1 week postoperatively using carbon dioxide (CO_2_) inhalation, followed by a physical method (opening the chest cavity) to confirm the death of the animal. These methods of euthanasia are consistent with the recommendation of the Panel on Euthanasia of the American Veterinary Medical Association (AVMA). Then place the animal in the supine position to prepare for dissection. Clean the abdominal skin by scrubbing the area with Povidone-Iodine and 70% of ethanol in three alternating cycles.
2. Make a midline incision of the abdominal skin before removing the abdominal organs to obtain visual access to the lumbar spine. Carefully dissect the lumbar spine while keeping the vertebral body and IVD intact.

Note: All steps for the tissue collection should be performed in a biosafety cabinet to maintain sterile conditions for cell isolation.

The detailed methods are described below.

#### 2.2.1 Preparation of Lumbar Bone Marrow Cells

**Figure 1:**
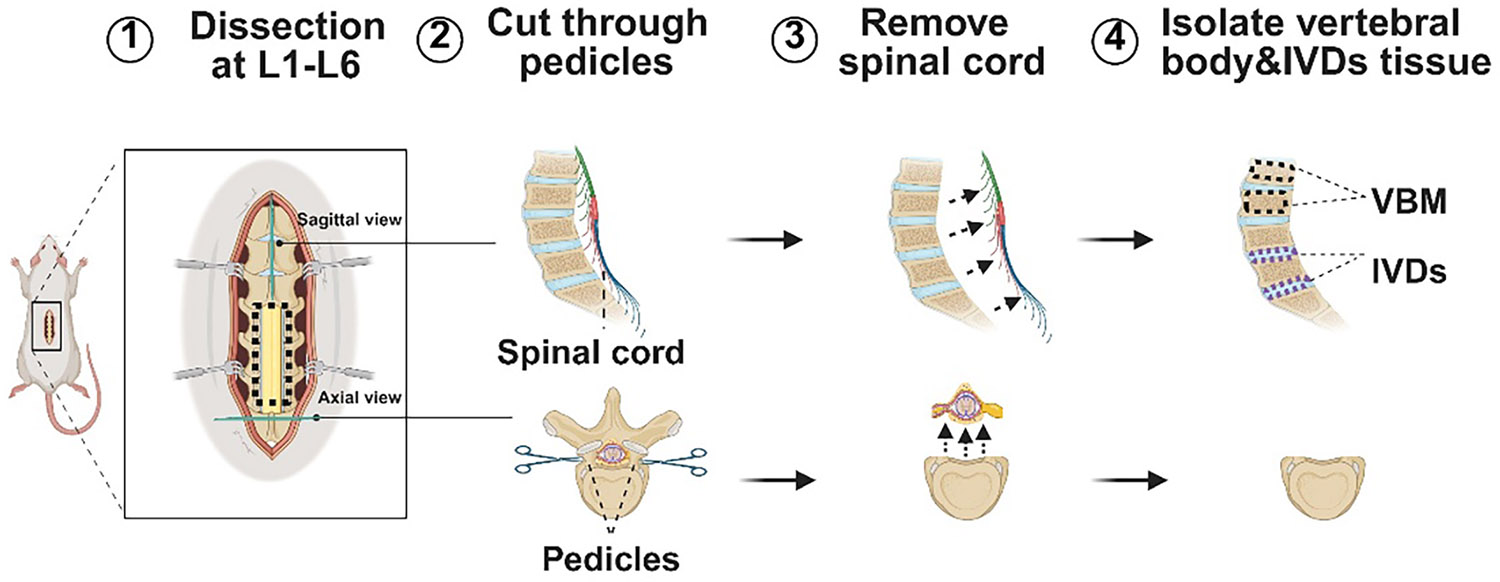
A flow chart of harvesting lumbar vertebral body and IVD tissues from the spine of mice (Created in https://BioRender.com). IVD: Intervertebral Disc; VBM: Vertebral Bone Marrow.

**Figure 2:**
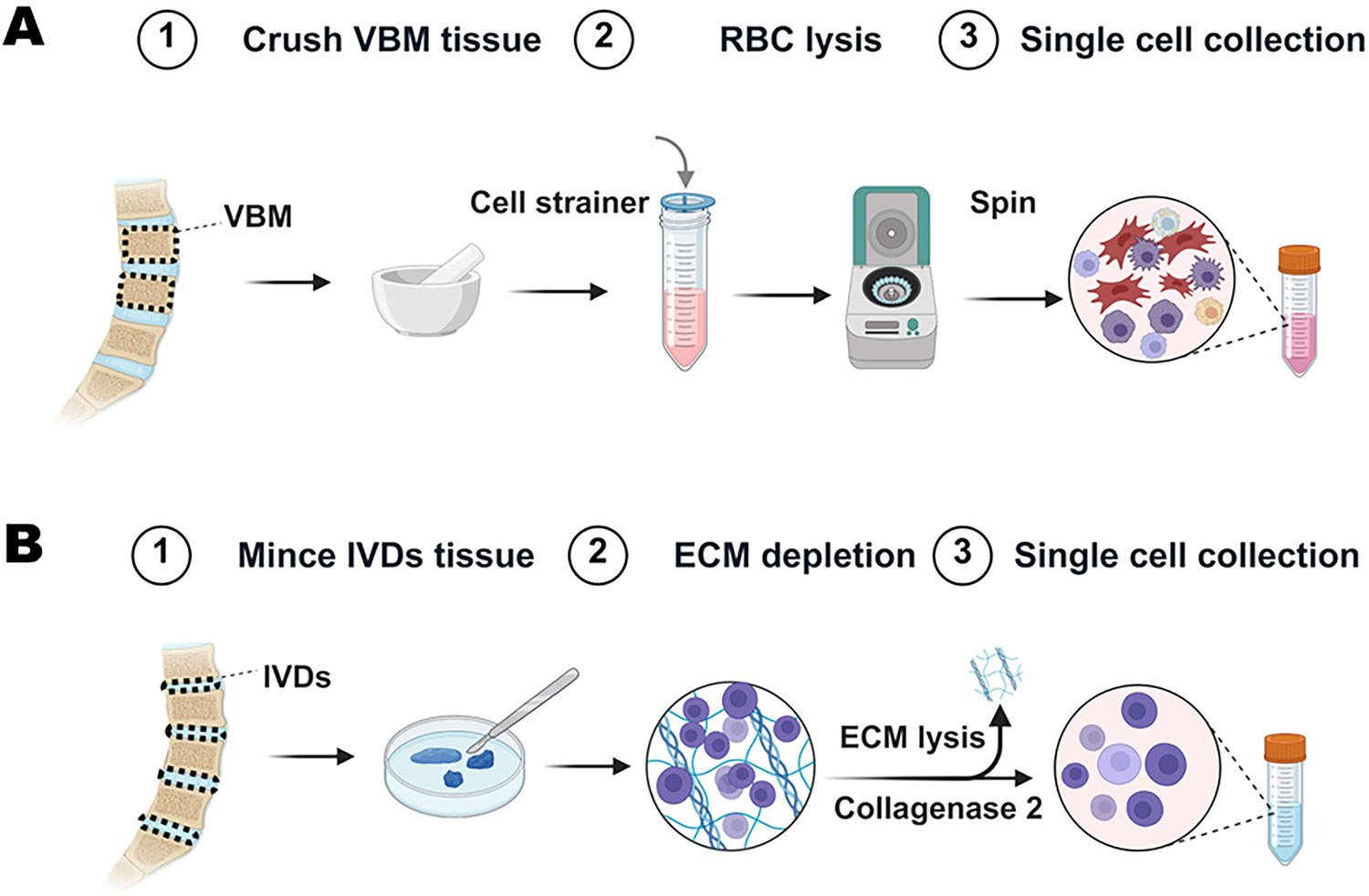
A flow chart of simultaneous single-cell collection from VBM (**A**) and IVD (**B**) tissues (Created in https://BioRender.com). ECM: Extracellular Matrix; RBC: Red Blood Cell.

1. Sterilize the biosafety cabinet, mortar and pestle, and supplies by ultraviolet (UV) for 30 min.
2. Clean tissues off the spine using sterilized scissors. Carefully harvest the IVD tissue under a dissecting microscope to avoid compromising the nearby vertebral body and bone marrow.
3. Hold the whole spine with tweezers, longitudinally cut through the pedicles of the lumbar spine, and open to spread apart the sides of the spine and expose the spinal cord (SC).
4. Gently detach the SC from the lumbar canal and remove it, making sure to prevent contamination from neurons and inflammatory cells around the SC (Fig. 1).
5. Carefully dissect the lumbar vertebrae at each IVD level from L3–L4 to L6–S1 using a scalpel blade, remove all IVD tissues to dissect out vertebral bodies of L4, L5 and L6, place them into mortar and add 5–10 mL of Roswell Park Memorial Institute 1640 Medium (RPMI 1640: SAPRA Biomedicals, SB-RPMI-002, Blacksburg, VA, USA) with 10% fetal bovine serum (FBS: Gibco, A5256701, Grand Island, NY, USA). Use a sterile pestle to crush the tissue.
6. Filter the cells on a 70 μm cell strainer that is placed on a 15 mL tube. Gently press the crushed bone marrow through the cell strainer with the plunger of a 3 mL syringe and rinse the strainer with another 3 mL of RPMI 1640 (SAPRA Biomedicals, SB-RPMI-002).
7. Spin the cells (500× *g*, 5 min, room temperature (RT/22°C) and resuspend the pellet in 5 mL of RBC lysis buffer (Abcam, ab204733, Cambridge, MA, USA). Incubate for 5 min at RT and stop the reaction by diluting the lysis buffer with 10 mL of phosphate-buffered saline (PBS).
8. Spin the cells (500× *g*, 5 min, 22°C), discard supernatant, and resuspend the pellet in 1 mL of PBS with 2% FBS (Gibco, A5256701, Grand Island, NY, USA). Refilter the suspension using a 30 μm cell strainer into a new 15 mL tube to remove coagulated cells (Fig. 2A). Count the number of live cells on a hemocytometer using the method of Trypan blue exclusion.

#### 2.2.2 Preparation of Intervertebral Disc Cells

1. Carefully dissect the lumbar spine and detach IVD tissue from vertebral bodies at four levels from L2-3 to L5-6 under a dissection microscope.
2. Mince the IVD tissue (including annulus fibrosus [1], nucleus pulposus, and endplate) into 0.5 mm^3^ pieces to enhance enzyme penetration.
3. Prepare the enzyme solution by dissolving collagenase type II (3 mg/mL) (Gibco, 17101015) in Dulbecco’s Modified Eagle Medium (DMEM: Corning Cellgro, 10-017-CV, Manassas, VA, USA) containing 4.5 g/L glucose and L-glutamine.
4. Transfer minced tissue into a 15 mL tube and add 8 mL of the pre-warmed enzyme solution (calculate with 2 mL/disc) and incubate at 37°C, gentle shaking (100–120 rpm) for 4–5 h.
5. Stop digestion by adding 800 μL of FBS (Gibco, A5256701) or 8 mL of PBS to neutralize the enzymes.
6. Filter the cell suspension through a 30 μm strainer into a fresh tube to remove debris.
7. Centrifuge at 500× *g* for 10 min at 22 °C to pellet cells. Discard the supernatant and resuspend the cell pellet in Phosphate-Buffered Saline (PBS: Cytiva, SH30256.02, Logan, UT, USA) +2% FBS (Fig. 2B).
8. Count cells using a hemocytometer or automated cell counter with Trypan Blue staining.

#### 2.2.3 Cell Allocation

1. Label tubes with sample ID, type of tissues, and the designated antibody panel. Two antibody panels are used in this protocol: Myeloid subset panel (extracellular staining) and T-cell subset panel (extracellular- and intracellular staining).
2. Provide at least 2.5 × 10^5^ cells per tube using the respective single cell suspensions.
3. For each panel and tissue type, dispense 100 μL of the 1 × 10^7^ cells/mL suspension for the unstained sample and a control with only 7-AAD dye that has been clearly marked, with 200 μL of the 1 × 10^7^ cells/mL suspension for all experimental tubes.

#### 2.2.4 Myeloid Subset Panel

1. Count Cells on Thermo Fisher Scientific Attune^™^ NxT (Thermo Fisher Scientific Inc., Waltham, MA, USA) using Propidium Iodide to exclude dead cells.
2. Dispense 100 μL of the 1 × 10^7^ cells/mL suspension for the unstained and the 7-AAD only tubes. Use the remaining cells in the all-antibody stain tube.
3. Stain the all-antibody tube with the extracellular markers. The staining buffer is Phosphate Buffered Saline (Cytiva, SH30256.02, Logan, UT, USA) supplemented with 2% heat inactivated FBS (Corning, 35-011-CV, Glendle, AZ, USA). The staining incubation was at 4°C for 25 min in the dark.
4. Wash with PBS wash buffer containing 10% FBS (Corning, 35-011-CV).
5. 5 μL of 7-AAD (Invitrogen, 00-6993-50, Waltham, MA, USA) was added to the appropriate tube 5 min before running on the flow cytometer. Incubate in the dark.
  Note: Treat single color reference controls just like the samples. The single color controls contain 1 drop of the Cytek beads +1 μL of antibody per single color control. Incubate at 4°C for 25 min in the dark.

#### 2.2.5 T Cell Subset Panel

1. Count Cells on Thermo Fisher Scientific Attune^™^ NxT (Thermo Fisher Scientific Inc., Waltham, MA, USA) using Propidium Iodide to exclude dead cells.
2. Dispense 100 μL of the 1 × 10^7^ cells/mL suspension for the unstained and the Zombie Aqua only tubes. Use the remaining cells in the all-antibody stain tube.
3. Dilute the Zombie Aqua 1:1000 in PBS, add 50 μL in Zombie Aqua tube, add 50 μL in all tubes, incubate 15 min at RT in the dark.
4. Wash with 1× wash buffer containing serum.
5. Stain all tubes with extracellular markers. Incubate at 4°C for 25 min.
6. Wash with wash buffer containing serum.
7. Add 1 mL of the True-Nuclear^™^ 1× Fix Concentrate to each tube, vortex, and incubate at RT in the dark for 45 min.
8. Add 2 mL of the True-Nuclear^™^ 1× Perm Buffer to each tube. Without washing, add 6.5 mL of 1X Perm Buffer for each sample of tube.
9. Centrifuge tubes at 300× *g* at RT for 5 min and discard the supernatant.
10. Add 2 mL of the True-Nuclear^™^ 1× Perm Buffer to each tube.
11. Centrifuge tubes at 300× *g* at RT for 5 min and discard the supernatant.
12. Resuspend the cell pellet in 100 μL of the True-Nuclear^™^ 1× Perm Buffer.
13. Add the appropriate amount of fluorochrome-conjugated antibody diluted in True-Nuclear^™^. 1× Perm Buffer for detection of intracellular antigen(s) to each well and incubate in the dark at room temperature for at least 30 min.
14. Add 2 mL of the True-Nuclear^™^ 1× Perm Buffer to each tube.
15. Centrifuge tubes at 300× *g* at RT for 5 min and discard the supernatant.
16. Add 2 mL of cell staining buffer.
17. Centrifuge tubes at 300× *g* at room temperature for 5 min and discard the supernatant.
18. Resuspend in 0.5 mL cell staining buffer, then acquire the tubes on a flow cytometer.
  Note: Treat reference controls just like the samples. Use 1 μL of antibody + Cytek Beads. For each antibody, the optimal concentration needs to be determined by performing a dose titration curve. Concentration between antibodies can differ drastically. For instance, CD3 and CD80 were used with a dilution factor of 1:1, while CD11b and CD4 were used with a dilution factor of 1:6400. When titrating the antibody concentration, use the same number of cells that will be used during the experiments.

**Figure 3:**
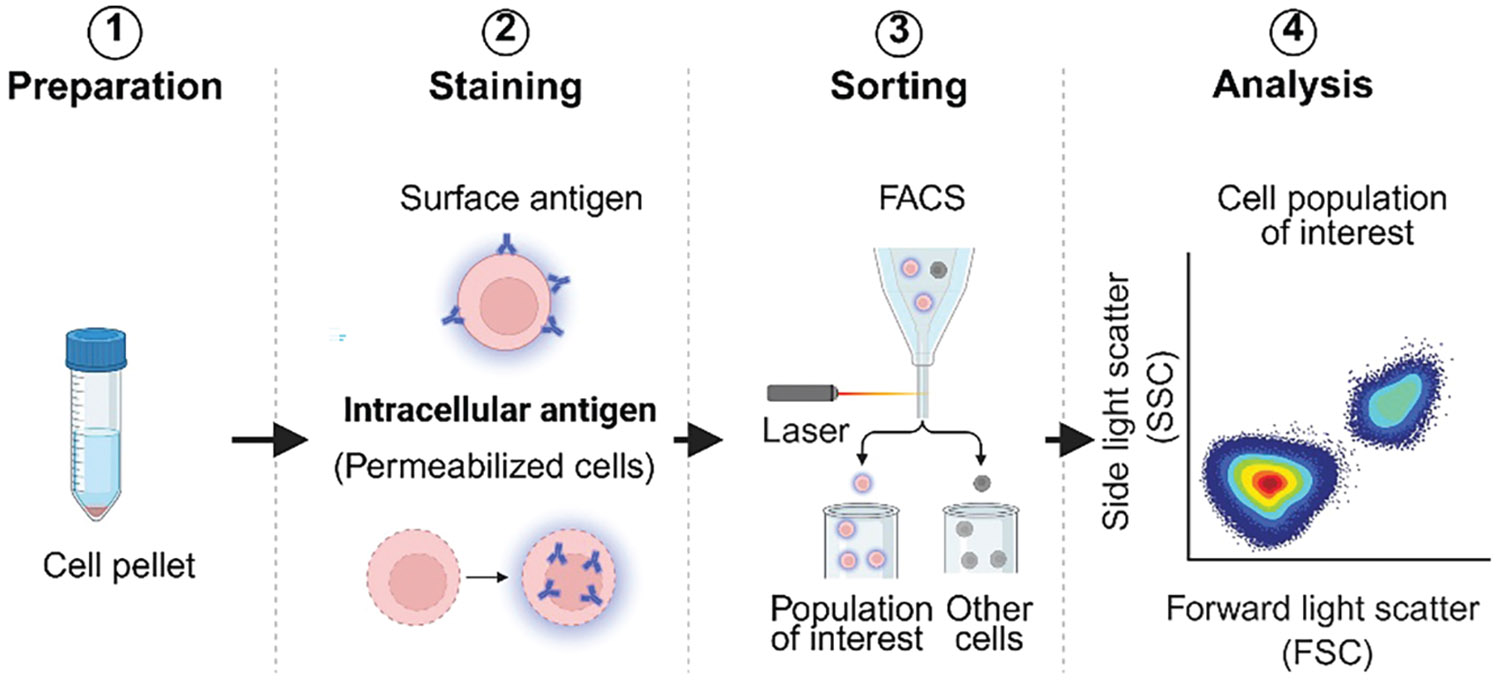
A workflow of cell preparation, staining, sorting, and FCM analysis (Created in https://BioRender.com). FCM: Flow Cytometry; FACS: Fluorescence-Activated Cell Sorting.

The step-by-step workflow of sample preparation for FCM is presented in Fig. 3.

#### 2.2.6 Compensation, Appropriate Controls, and Gating

1. Setting up the experiment
  - Once the optimal antibody concentration has been determined, run unstained and single-stained controls to adjust for spectral overlap. Note: Run reference controls using compensation beads except for the unstained and live/dead dye controls.
  - Determine the optimal Forward Scatter Area (FSC-A) voltage and Side Scatter Area (SSC-A) voltage to detect the leukocyte population in unstained controls of each tissue type. Note: The fixation and permeabilization process alters the dimensions of the cell. Thus, the FSC-A and SSC-A voltages for the Monocyte Subset Panel and T Cell Subset Panel may differ. Find the optimal voltages for the T Cell Subset Panel, use cells that have been single-stained with CD3, and back gate towards the leukocyte populations while adjusting the FSC-A and SSC-A values.
2. Gating strategy
  - Set up a primary gate on the leukocyte population once the optimal FSC-A and SSC-A voltage has been determined. These cells were defined as CD45^+^ in the myeloid samples or CD3^+^ in the T cell samples. Note: Prior to each experiment, calibrate the cytometer using calibration beads as per the manufacturer’s instructions and run unstained beads. Leukocyte populations of different time points should have comparable FSC and SSC properties (slight differences between tissue types are expected and normal). If FSC and SSC varies considerable trouble shoot the cytometer and sample generation.
  - Exclude doublets: Plot FSC-A (*y*-axis) and FSC-H (*x*-axis). The singlet gate is based on the proportion between Height values and Area values within the same type of scatter. Doublet cells have double the area but only 1× Height.
  - Exclude dead cell: Plot FVS510 (viability stain) (*x*-axis) and FSC-A (*y*-axis). Dead cells will appear as positive events, thus gate on live cells. Note: True negative cells will be visible in unstained controls. Thus, adjust this gate for each set of samples when running the unstained controls prior to stained samples. Further gating depends on the antibody panel and cell type that is investigated.

**Figure 4:**
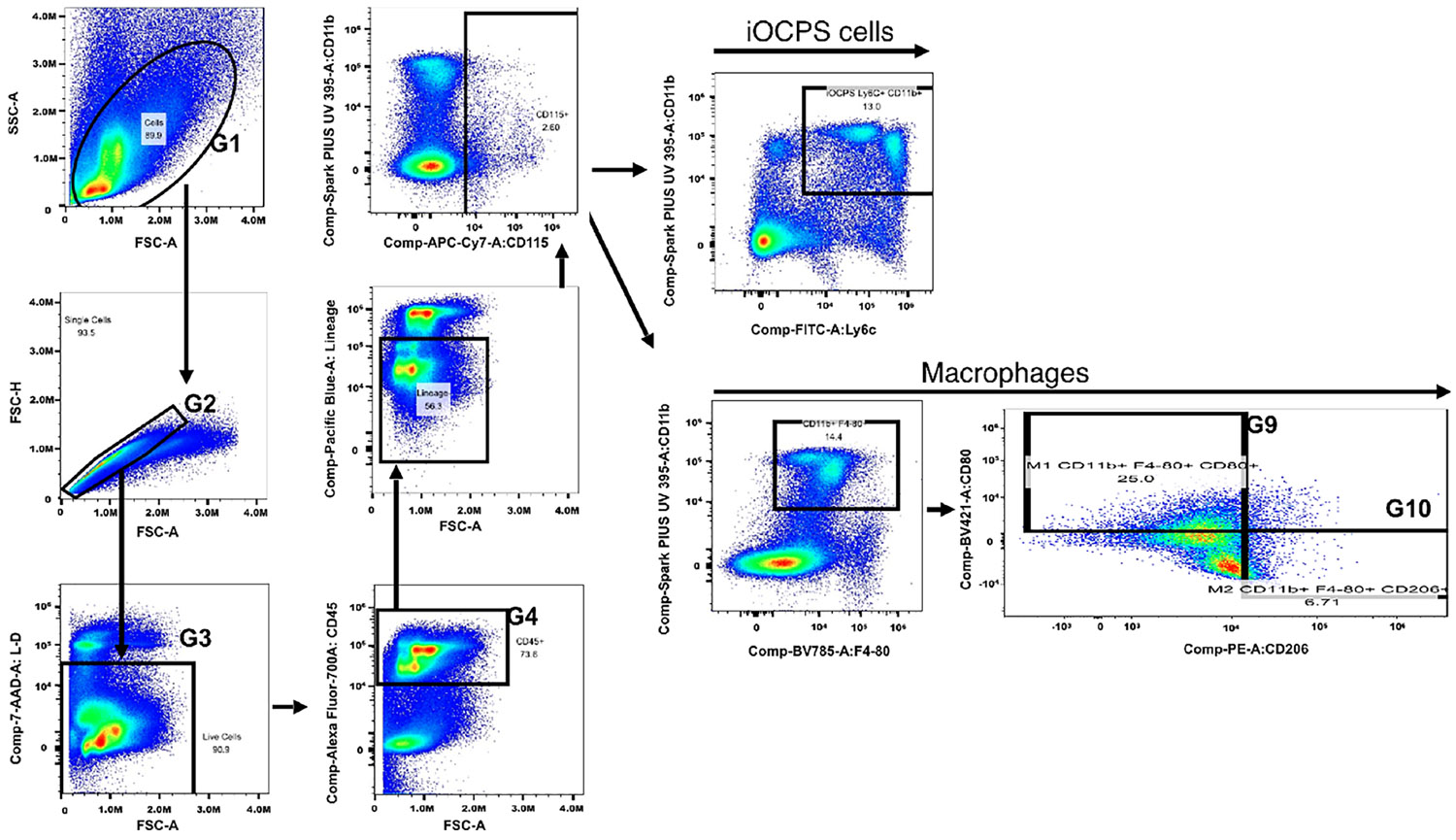
Flow cytometry hierarchical gating strategy using extracellular staining to identify macrophages and their phenotypes. Myeloid and lymphoid cells are primarily identified using a forward/side scatter (FSC-A and SSC-A) dot plot (G1). Thereafter, singlets are detected using FSC-A and FSC-H (G2), and then live cells are selected (G3). From live cells, all pan-leukocytes (G4) were gated on using CD45, followed by selection of lineage-negative (Lin ^−^) cells/undifferentiated progenitor cells (G5). Within the Lin^−^ gate, CD11b^+^ cells (G6) were identified. Cells from G5 are further classified using Ly-6G to identify iOCPS cells (G7) and F4/80 for macrophages (G8). Macrophages are further classified into their phenotypes using CD80 and CD206. M1: CD80^+^/CD206^−^ (G9); M2: CD80^−^/CD206^+^ (G10). FSC-A: Forward Scatter Area, SSC-A: Side Scatter Area, iOCPS: Immature Osteoclast Progenitor cells.

**Figure 5:**
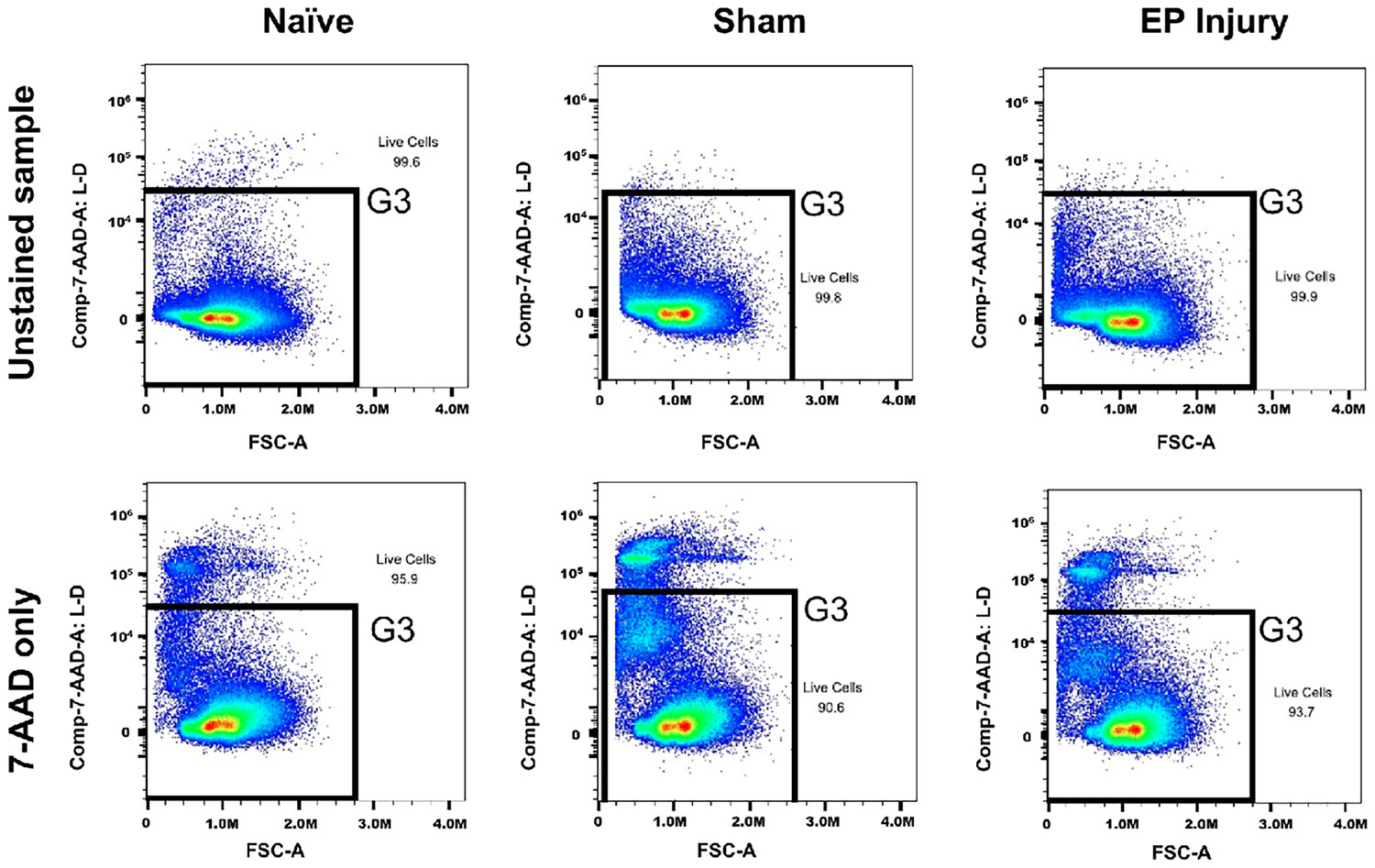
Sample data illustrating appropriate controls in the Myeloid panel. Lumbar bone marrow tissue was harvested from Naïve, Sham, or EP injury groups at 1 week after surgery. A single cell suspension was stained using extracellular surface markers. During each experiment, an unstained sample and a single-color dye (7-AAD) control were used to determine true negatives for the dead/alive stain and to set gates (Control). Setting of gates using unstained cells and single-color dye is shown in the panel. EP: Endplate.

**Figure 6:**
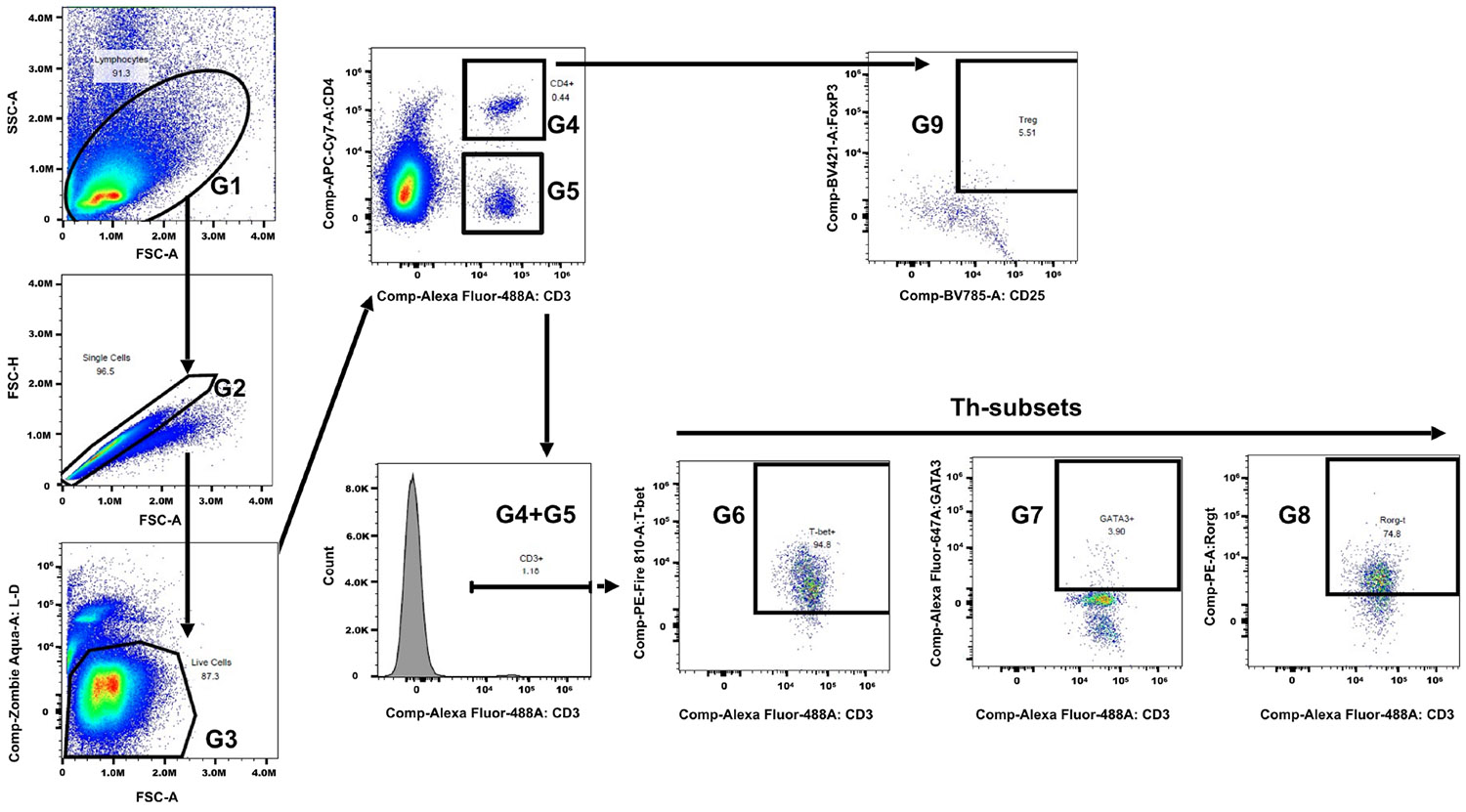
Flow cytometry hierarchical gating strategy using both extra- and intracellular staining to identify T-cells and their subsets. T cells are primarily identified using a forward/side scatter (FSC-A and SSC-A) dot plot (G1). Thereafter, singlets are detected using FSC-A and FSC-H (G2), and then live cells are selected (G3). Cells from G3 are classified using CD3 to identify T cells, followed by CD4 expression to distinguish CD3^+^CD4^+^T cells (G4) and CD3^+^CD4^−^ T-cells (G5). T-helper cells are classified into Th-1 (CD3^+^/T-bet^+^ (G6)), Th-2 cells (CD3^+^/GATA3^+^ (G7)) and Th-17 cells (CD3^+^ RORgt^+^ (G8)) using intracellular markers. In addition, T-regulatory cells (CD4^+^CD25^+^/FoxP3^+^ (G9)) are identified using intracellular markers.

**Figure 7:**
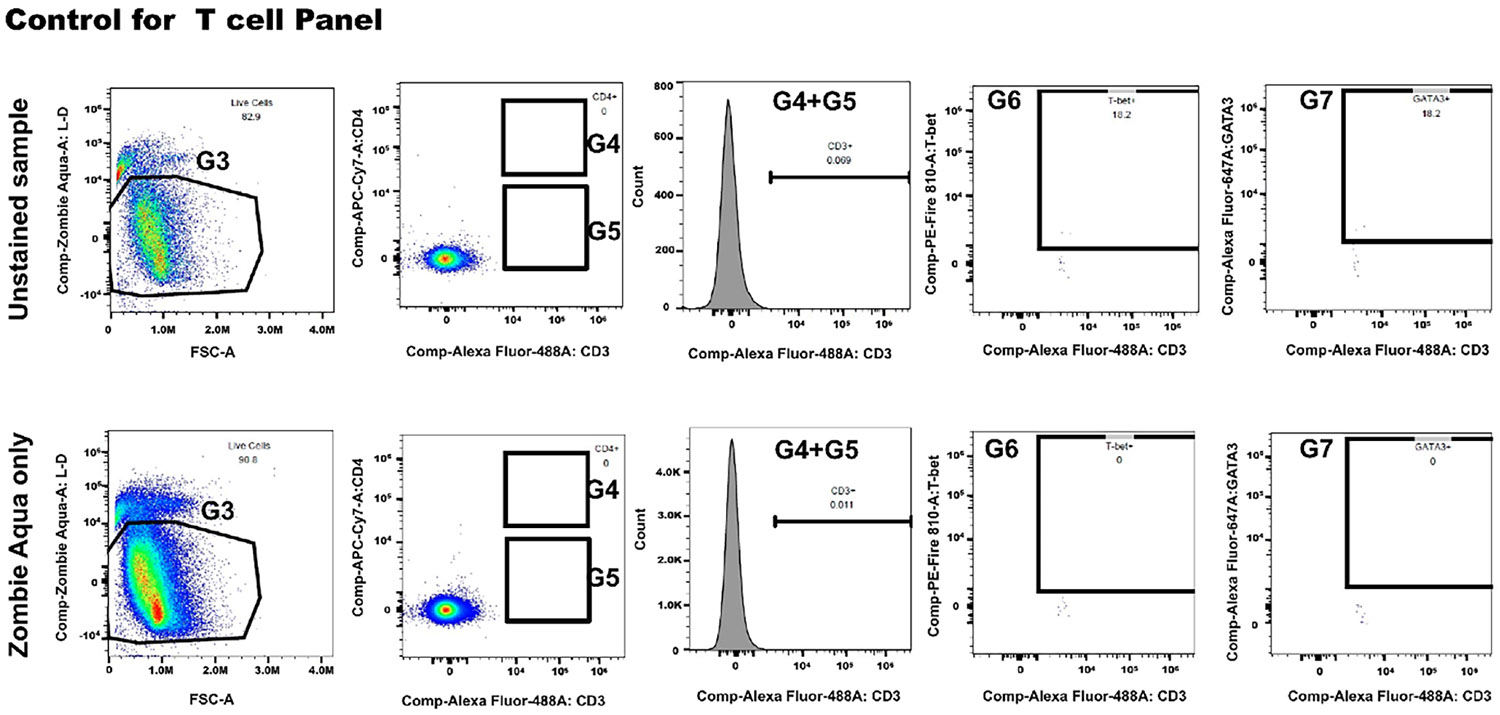
Sample data illustrating appropriate controls in the T-cell panel. Lumbar bone marrow tissue was harvested either from mice in the Sham or EP injury groups at 1 week after surgery. A single cell suspension was stained using both extracellular surface and intracellular markers. An unstained sample and a single-color dye (Zombie Aqua) control were used to determine true negatives for the dead/alive stain and to set gates (Control).

Gating strategies and control for myeloid subset panel can be found in Figs. 4 and 5, respectively. Gating strategies and control for T-cell subset panel can be found in Figs. 6 and 7, respectively.

#### 2.2.7 Flow Cytometry Analysis

The antibodies and their specifications used for FCM analysis are summarized in Table 1.

### 2.3 Statistics

1. Perform statistical analyses using data from quantitative FSC measurements.
2. Determine the significance of differences between study groups using one-way ANOVA, followed by a post-hoc test (Tukey) using Prism (GraphPad, LaJolla, CA, USA), Version 8.0.2. A *p*-value of <0.05 was considered statistically significant.

### 3. Results

At 1-week following surgical injury, significantly increased macrophage numbers and M2a polarization response were observed in VBM samples from the sham and EP injury groups, while the ratio of M2a/total macrophage was significantly increased and the ratio of M1/M2a was significantly decreased in IVDs from the sham and EP injury groups. A significantly increased Treg cell response was detected in VBM samples from the EP injury group, but not the sham group.

Cell recovery rate after enzymatic digestion for the T cell panel is presented in Supplementary Table S1. Cell recovery rates after enzymatic digestion and CD45 positivity for the myeloid panel are presented in Supplementary Table S2.

**Figure 8:**
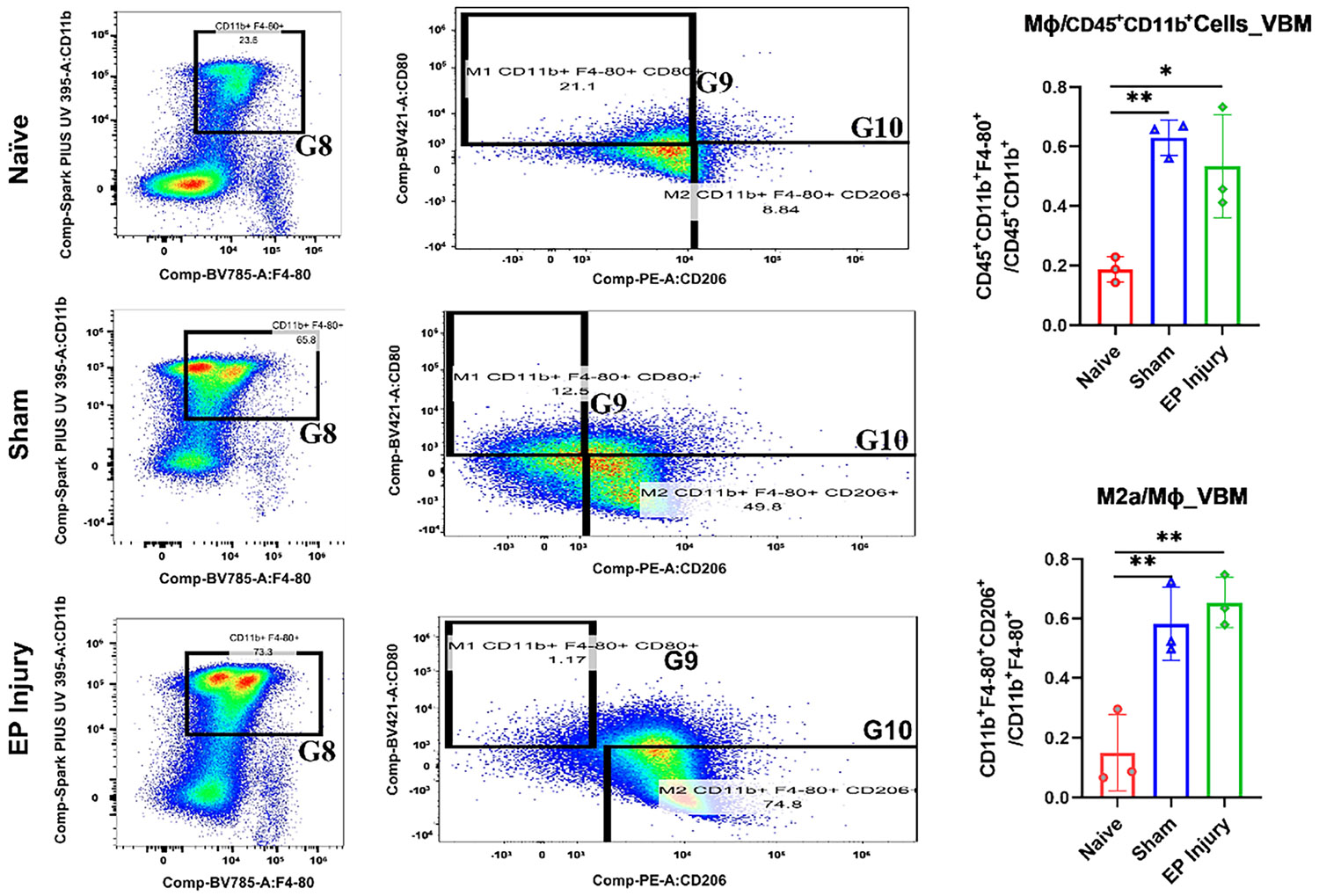
Extracellular staining of monocytes/macrophages isolated from the lumbar bone marrow of mice. Tissue samples were collected either from Naïve mice or from Sham and EP injury groups at 1 week after surgery. Gate information (G8–G10) utilized here is the same as that described in Fig. 4 legend. Sample data shows that all subsets can be reliably identified, and statistical differences can be detected among the three groups. N = 3, *p < 0.05, **p < 0.01.

**Figure 9:**
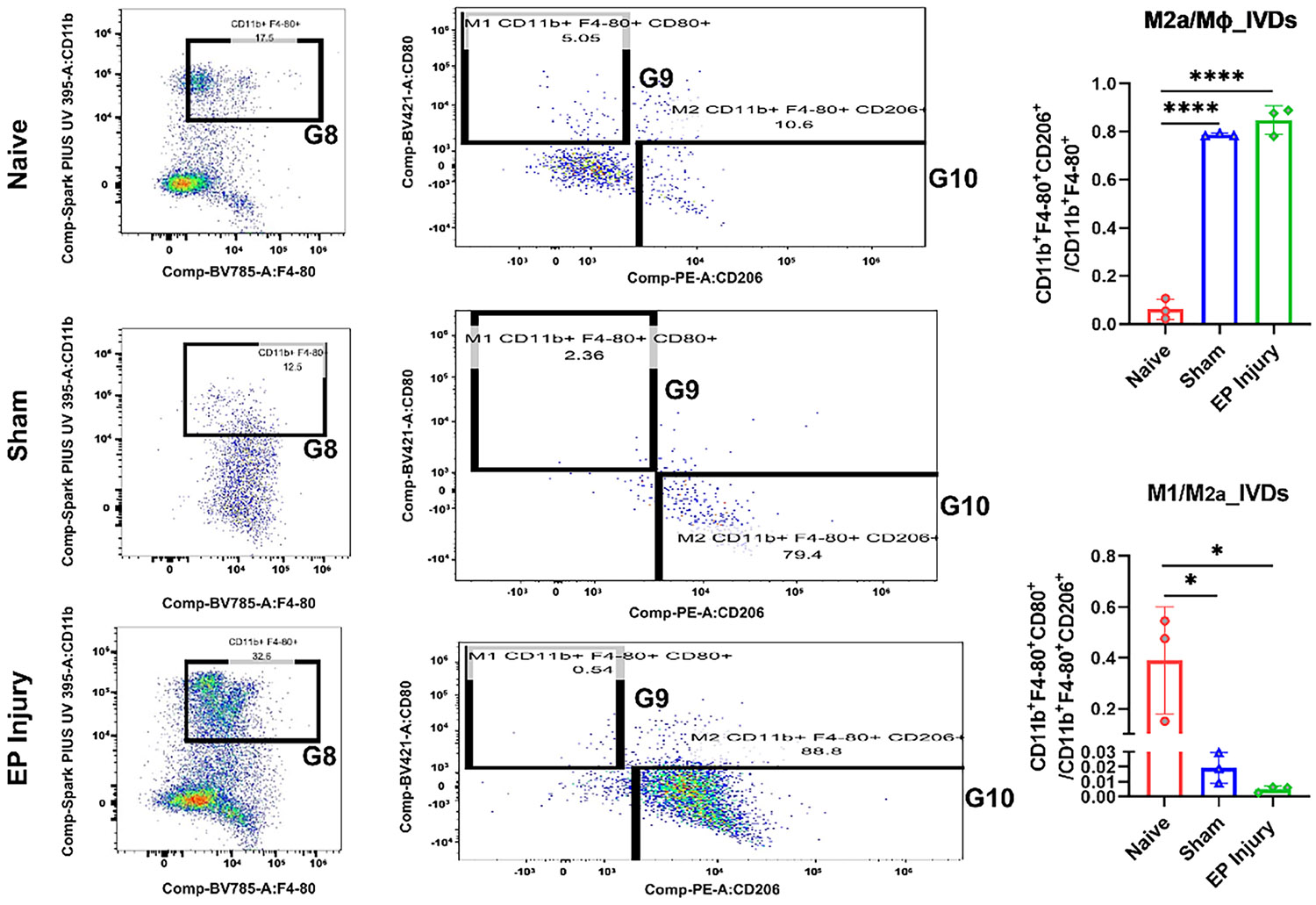
Extracellular staining of macrophages isolated from the lumbar IVDs of mice. Tissue samples were collected either from Naïve or from Sham and EP injury groups at 1 week after surgery. MΦ = total number of macrophages. N = 3, *p < 0.05, ****p < 0.0001.

**Figure 10:**
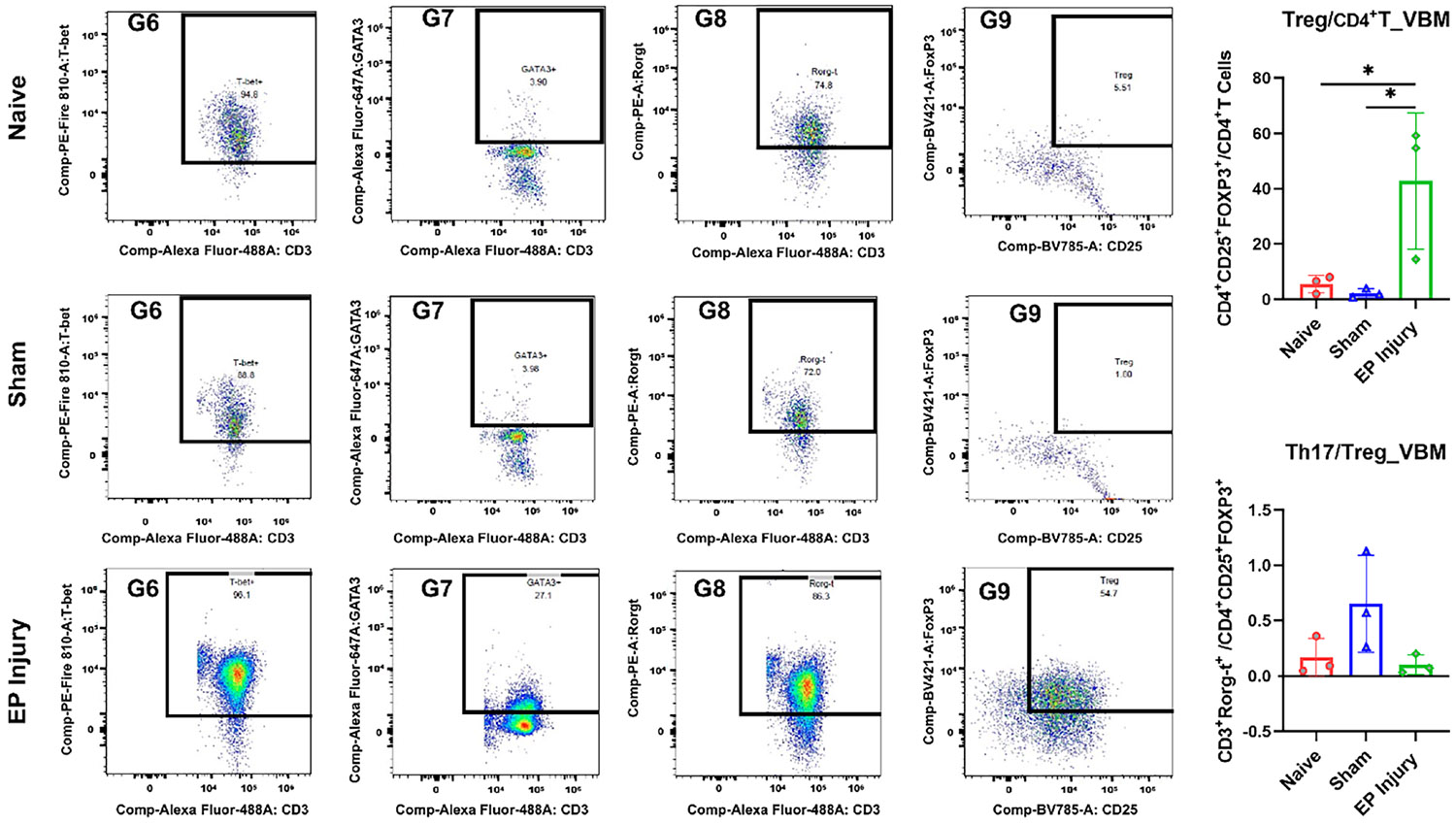
Extracellular and intracellular staining of T-cells isolated from lumbar bone marrow tissue of mice. Tissue samples were collected either from Naïve or from Sham and EP injury groups at 1 week after surgery. Further information (G6–G9) utilized here is the same as that described in Fig. 6. FCM data show that all subsets can be reliably identified, and differences can be seen between the groups. N = 3, *p < 0.05.

Representative results from FSC analysis of the myeloid subset panel and T-cell subset panel are presented in Figs. 8-10.

## 4 Discussion

The importance of identifying and understanding the immune-inflammatory cell response during acute IVD injury and disc herniation has been widely studied [25,28-30], yet the majority of studies concentrate only on IVDs tissue, rather than integrating VBM analysis. This article is the first methodological report on FCM analysis of immune cells isolated from both IVD and adjacent VBM to study the local immune-inflammatory responses to EP microfracture in mice. First, the mouse model of EP microfracture has not been reported previously. Second, using FCM to detect immune cells in both IVD and VBM of mice after EP microfracture is a novel approach. Third, although the data used in this article are focused on the early timepoint following EP microfracture, the methods for tissue harvest and cell isolation allow for longitudinal follow-up observations to investigate the dynamic alterations of immune-inflammatory responses to EP injury at multiple timepoints in any animal models. In contrast, FCM analyses of both IVD and VBM cells from live human patients with MCs were usually limited to a single timepoint [23]. Thus, the methodology presented here can significantly advance the mechanistic cellular study of MCs and IVD degeneration.

Previous studies found that EP microfracture injury can cause MC1-like changes in all injured lumbar specimens [19], increased infiltration of CD68^+^ macrophages in the EP and vertebrae, and IVD degeneration at postoperative 8-weeks in rats [20]. In the current study, at postoperative 1-week, we further found that sham and EP injured mice all displayed significant M2a polarization response in both VBM and IVD, with significantly increased ratio of M2a/total macrophage. In addition, a significantly decreased ratio of M1/M2a was detected within IVD for both the Sham and EP injury groups. Interestingly, *in vitro* co-culture of bone marrow-derived macrophages and nucleus pulpous cells resulted in a 60% polarization of macrophages toward the M2 phenotype [31]. The enhanced macrophage and Th17 cells in human herniated NP regions suggest a correlation of macrophage and Th17 cells with ruptured NP mediated immune-inflammatory response [25]. The pro-inflammatory ‘polarization’ of macrophages (M1) is likely induced by Th1 cytokines such as IFN-γ. In contrast, the anti-inflammatory ‘polarization’ of macrophages (M2) is promoted by Th2 cytokines such as IL-4 [27]. It seems to indicate some correlations between subpopulations of T helper balance shift and M1/M2 phenotypic polarization, and the polarization of CD4^+^Th might be involved in the ruptured NP mediated immune-inflammatory response through the phenotypic shift of macrophages [26,27]. In the current study, EP injured mice exhibited a significant Treg cellular response in VBM, while sham-injured mice lacked this change, suggesting that a crosstalk and immune response of VBM and NP occurred following EP rupture, since the Treg cells play a crucial role in suppressing autoimmune responses and promoting immune tolerance [32].

Animal models of EP injury are required to include an appropriate control group(s) when elucidating the pathogenic mechanism of immune-inflammatory responses and screening potential treatments for pain sources. IVD degeneration can be classified into EP-driven and AF-driven phenotypes [4,33,34]. The AF-driven phenotype has been widely studied as a traditional focus of investigation in murine models [35-38], while the pivotal role of the EP-driven phenotype is gaining increased emphasis [17,19,39]. However, all the above animal models of IVD degeneration exhibit their own advantages and inherent limitations because surgical trauma and its associated inflammatory response might serve as a confounder when investigating the immune-inflammatory response in the spinal tissues, especially at the early stage following the surgical intervention [40]. Thus, this study included a naïve group as a baseline control and a sham group as a vertebral bone trauma control. These control groups have helped us more accurately analyze the EP injury elicited immune-inflammatory cellular responses by FCM.

The volume of cells in the mouse IVD is low due to the nature of low cellularity and the small size of IVDs. The tissue dissection process is technically challenging and must be done under a microscope. In order to obtain the required number of IVD cells for FCM analysis, cells isolated from 2 or more lumbar IVDs of the same animal group can be pooled to form a single IVD cell sample for FCM analysis. The digestion process and choice of enzymes for IVD samples should be optimized to fully digest the extracellular matrix (ECM).

Antibody selection methods for FCM have been described and tested in this study. Our results have confirmed reliable determination of various subsets from both myeloid- and T-cells within the lumbar VBM and the adjacent IVD in the mouse model of MCs. The protocol presented here can easily be adopted to investigate distinct immune cell types by designing a cocktail of antibodies spectrum and to explore the cellular mechanism for spinal inflammation associated with EP injury and painful IVD degeneration and MCs. When piloting antibodies for cells from other tissue types, it is crucial to investigate the specificity of each antibody, as the expression of surface markers of immune cells may vary in different tissues. In addition, when exchanging antibodies, it is necessary to generate a dose titration curve to get the optimal concentration of specific antibodies and repeat the compensation process to address changes in spectral overlap.

A potential limitation of this protocol lies in the selection of markers for cell identification due to the wide array of available markers and the lack of consensus across studies in defining specific cell populations [41]. Another limitation is that when adapting the protocol to other tissues or species, pilot testing of each step is crucial to validate the effectiveness of the methods. The current protocol may be translatable to human samples with appropriate adjustments; however, antibodies need to be reselected and tested since the surface markers of human and murine immune cells exhibit distinct characteristics.

Despite these adjustable limitations, the methodology of tissue dissection, cell isolation, and antibody selection for FCM analysis proves to be specific for distinguishing both macrophage cells and T-cell subsets at the single-cell level in mice. In particular, the current protocol describes a reliable and reproducible strategy that can identify and quantify the alterations of immune cells within the VBM and adjacent IVD tissue during the early phase of MCs. This methodology may help characterize the immune-inflammatory responses to EP injury in other species. Furthermore, this study may assist in identifying risk factors for back pain and contribute to screening therapeutics of immunomodulatory drugs.

## 5 Conclusion

In conclusion, this protocol provides reproducible and reliable methods for spinal tissue dissection, cell isolation, and antibody selection for FCM analysis using both extra- and intracellular staining assays to identify macrophage and T cell subsets within the lumbar VBM and IVD tissues in a mouse model of surgically induced EP microfracture.

## Supplementary Material

Supplementary Table 1

Supplementary Table 2

Supplementary Materials: The supplementary material is available online at https://www.techscience.com/doi/10.32604/biocell.2026.074572/s1.

## Figures and Tables

**Table 1:** List of antibodies used for flow cytometry.

Antibody	Host Species/Isotype	Source		Catalog Number	Amount/Sample
		Vendor	Location (USA)		
CD45	Rat/IgG2b	BioLegend	San Diego, CA	147715	0.25 μg
Ly-6C	Rat/IgG2c	BioLegend	San Diego, CA	128005	0.25 μg
CD11b	Rat/IgG2b	BioLegend	San Diego, CA	101238	0.25 μg
CD90.2 (Thy1.2)	Rat/IgG2b	BioLegend	San Diego, CA	105323	1 μg
CD45R/B220	Rat/IgG2a	BioLegend	San Diego, CA	103230	1 μg
TER-119	Rat/IgG2b	BioLegend	San Diego, CA	116231	0.25 μg
Ly-6G	Rat/IgG2a	BioLegend	San Diego, CA	127611	1 μg
F4/80	Rat/IgG2a	BioLegend	San Diego, CA	123141	0.5 μg
CD115	Rat/IgG2a	BioLegend	San Diego, CA	135531	0.25 μg
CD80	Armenian Hamster/IgG	BioLegend	San Diego, CA	104725	0.25 μg
CD206	Rat/IgG2a	BioLegend	San Diego, CA	141705	0.5 μg
CD3	Rat/IgG2b	BioLegend	San Diego, CA	100212	0.25 μg
CD4	Rat/IgG2a	BioLegend	San Diego, CA	100525	1 μg
anti-T-bet	Mouse/IgG1	BioLegend	San Diego, CA	644839	1 μg
CD25	Rat/IgG1	BioLegend	San Diego, CA	102051	0.5 μg
anti-GATA3	Mouse/IgG2b	BioLegend	San Diego, CA	653809	0.125 μg
anti-mouse FOXP3	Rat/IgG2b	BioLegend	San Diego, CA	126419	0.5 μg
ROR gamma (t)	Rat/IgG1	Thermofisher	Waltham, MA	12-6981-82	0.25 μg

Note: FCM samples were measured on a Cytek Aurora flow cytometer (Cytek Biosciences, Fremont, CA, USA) and data collected by SpectroFlo Software (version 3.3; Cytek Biosciences, Fremont, CA, USA), and data analyzed using FlowJo software (version 10.10; Becton, Dickinson and Company, Ashland, OR, USA). Full name of the antibody abbreviations listed in Table 1. CD: Cluster of Differentiation; Ig: Immunoglobulin; Ly-6C: Lymphocyte antigen 6 complex locus C; TER-119 (gene name of Ly-76): Lymphocyte antigen 76; Ly-6G: Lymphocyte antigen 6 complex locus G; F4/80 (EMR1): EGF-like module-containing mucin-like hormone receptor 1; T-bet: T-box expressed in T cells; GATA3: GATA binding protein 3; FOXP3: Forkhead Box P3; ROR gamma: Nuclear receptor ROR-gamma.
