# Supplementary material for: Flow Cytometry Study of Immune Cell Subpopulations from the Mouse Vertebral Bone Marrow and Intervertebral Disc Following Endplate Microfracture
Source: Biocell. Author manuscript; Available in PMC 2026 Jul 7. (PMC13337323; doi:10.32604/biocell.2026.074572)
Supplement: Supplementary Table 1 [file NIHMS2176528-supplement-Supplementary_Table_1.docx]

|  | Lymphocytes/Single Cells Count | Lymphocytes/Single Cells/Live Cells Count | Lymphocytes/Single Cells/Live Cells/ Freq. of Parent |
| --- | --- | --- | --- |
| Naive 1-IVD | 25521 | 2685 | 10.50% |
| Naive 2-IVD | 163655 | 93790 | 57.30% |
| Naive 3-IVD | 94808 | 28520 | 30.10% |
| Naive 1-VBM | 239180 | 208698 | 87.30% |
| Naive 2-VBM | 392375 | 356675 | 90.90% |
| Naive 3-VBM | 705000 | 685000 | 97.10% |
| Sham 1-IVD | 18386 | 1965 | 10.70% |
| Sham 2-IVD | 73514 | 13863 | 18.90% |
| Sham 3-IVD | 105627 | 18825 | 17.80% |
| Sham 1-VBM | 185589 | 157185 | 84.70% |
| Sham 2-VBM | 510000 | 474000 | 92.90% |
| Sham 3-VBM | 346757 | 302476 | 87.20% |
| EP Injury 1-IVD | 124580 | 27991 | 22.50% |
| EP Injury 2-IVD | 108849 | 39333 | 36.10% |
| EP Injury 3-IVD | 71035 | 11548 | 16.30% |
| EP Injury 1-VBM | 336796 | 311343 | 92.40% |
| EP Injury 2-VBM | 635000 | 591000 | 93.10% |
| EP Injury 3-VBM | 546000 | 494000 | 90.30% |

**Supplementary Table 1. Cell recovery rate after enzymatic digestion for T cell panel**
