# Supplementary material for: Flow Cytometry Study of Immune Cell Subpopulations from the Mouse Vertebral Bone Marrow and Intervertebral Disc Following Endplate Microfracture
Source: Biocell. Author manuscript; Available in PMC 2026 Jul 7. (PMC13337323; doi:10.32604/biocell.2026.074572)
Supplement: Supplementary Table 2 [file NIHMS2176528-supplement-Supplementary_Table_2.docx]

|  | Single Cells Count | Single Cells/Live Cells /Count | Cells/Single Cells/Live Cells /Freq. of Parent | Cells/Single Cells/Live Cells/CD45+ /Count | Cells/Single Cells/Live Cells/CD45+ /Freq. of Parent |
| --- | --- | --- | --- | --- | --- |
| Naive 1-IVD | 142599 | 138920 | 97.4 % | 22613 | 16.3 % |
| Naive 2-IVD | 145018 | 136364 | 94.00% | 55928 | 41.00% |
| Naive 3-IVD | 68848 | 67576 | 98.20% | 8471 | 12.50% |
| Naive 1-VBM | 237862 | 222108 | 93.4 % | 186200 | 83.8 % |
| Naive 2-VBM | 495000 | 450000 | 90.90% | 330873 | 73.60% |
| Naive 3-VBM | 520000 | 471000 | 90.40% | 403000 | 85.60% |
| Sham 1-IVD | 199033 | 196485 | 98.70% | 12335 | 6.28% |
| Sham 2-IVD | 9711 | 8800 | 90.60% | 2902 | 33.00% |
| Sham 3-IVD | 179345 | 163968 | 91.40% | 36974 | 22.50% |
| Sham 1-VBM | 725000 | 669000 | 92.20% | 427000 | 63.90% |
| Sham 2-VBM | 379727 | 335000 | 88.20% | 289342 | 86.40% |
| Sham 3-VBM | 519000 | 486000 | 93.60% | 453000 | 93.30% |
| EP Injury 1-IVD | 182887 | 176139 | 96.30% | 30288 | 17.20% |
| EP Injury 2-IVD | 137019 | 133232 | 97.20% | 20480 | 15.40% |
| EP Injury 3-IVD | 154329 | 151423 | 98.10% | 12891 | 8.51% |
| EP Injury 1-VBM | 525000 | 489000 | 93.10% | 427000 | 87.30% |
| EP Injury 2-VBM | 498000 | 429000 | 86.20% | 340351 | 79.30% |
| EP Injury 3-VBM | 313894 | 250341 | 79.80% | 165232 | 66.00% |

**Supplementary Table 2. Cell recovery rate after enzymatic digestion and CD45 positivity rate for myeloid panel**
